# Supplementary material for: The Construction and Exploration of a Comprehensive MicroRNA Centered Regulatory Network in Foxtail Millet (Setaria italica L.)
Source: Front Plant Sci. 2022 May 6;13:848474. doi: 10.3389/fpls.2022.848474 (PMC9121102; doi:10.3389/fpls.2022.848474)
Supplement: Supplementary file 5 [file Data_Sheet_5.zip › Sit-miRN2548_Seita.5G109100.1_895_TPlot.pdf]

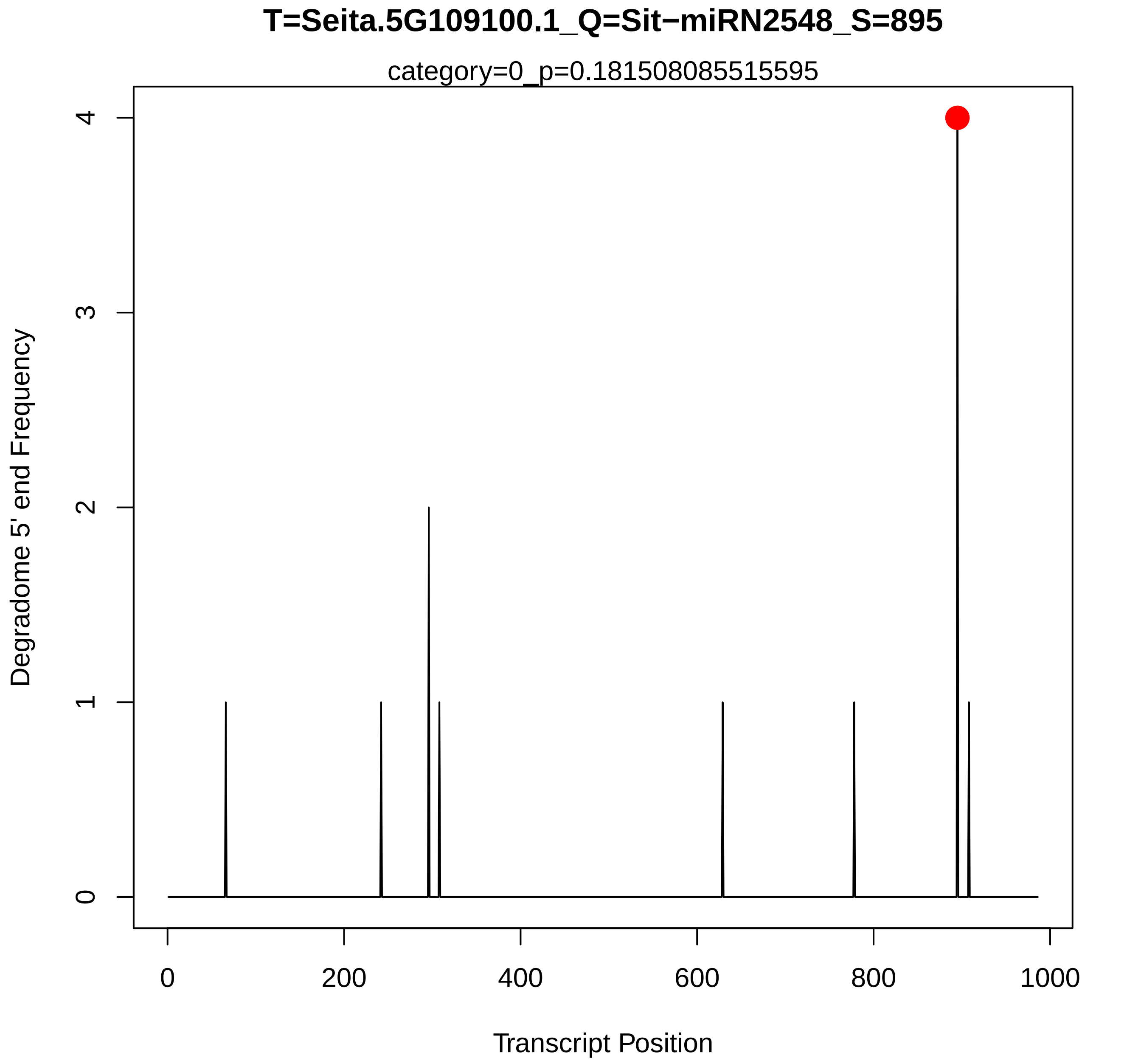

T=Seita.5G109100.1_Q=Sit−miRN2548_S=895
category=0_p=0.181508085515595
Degradome 5' end Frequency
4
3
2
1
0
0
200
400
600
800
1000
Transcript Position
